# Intake of residuals from Atlantic cod attenuated blood pressure increase but did not delay development of kidney damage in obese Zucker fa/fa rats

**DOI:** 10.29219/fnr.v66.8708

**Published:** 2022-10-11

**Authors:** Iselin Vildmyren, Åge Oterhals, Sabine Leh, Tor Andreas Samuelsen, Alfred Halstensen, Hans-Peter Marti, Oddrun Anita Gudbrandsen

**Affiliations:** 1 Dietary Protein Research Group, Department of Clinical Medicine, University of Bergen, Haukeland University Hospital, Bergen, Norway; 2 Nofima, Bergen, Norway; 3 Department of Pathology, Haukeland University Hospital, Bergen, Norway; 4 Department of Clinical Medicine, University of Bergen, Bergen, Norway; 5 Department of Clinical Science, University of Bergen, Bergen, Norway

**Keywords:** fish proteins, fish residuals, blood pressure, hypertension, kidney, obesity

## Abstract

**Background:**

Documentation of health effects of residuals after fish filleting may motivate both consumers and producers to increase the use of this under-utilised protein source.

**Objectives:**

The primary objective of the present study was to investigate the effects of a diet containing residuals from Atlantic cod (*Gadus morhua*) filleting on the development of high blood pressure in obese Zucker fa/fa rats, which spontaneously develop hypertension and proteinuria. The secondary objectives were to investigate any changes in kidney morphology, kidney function and organ damage, and to determine the potential inhibition of cod residuals on renin and angiotensin-converting enzyme (ACE) activities in vitro.

**Methods:**

Male rats were fed diets containing protein powder prepared from head, backbone and skin fraction (HBS, *n* = 6) from Atlantic cod as 25% of total protein with the remaining 75% as casein, or casein as the sole protein source (Control group, *n* = 6) for 4 weeks. Blood pressure was measured on day 0, 14 and 26. Kidneys were analysed morphologically, and markers for renal function and organ damage were analysed biochemically.

**Results:**

The HBS diet attenuated the blood pressure increase compared to the Control group, but kidney damage and dysfunction were similar between the two groups.

**Conclusion:**

A diet containing a protein powder consisting of HBS fraction from cod attenuated the blood pressure increase in obese Zucker fa/fa rats, without preventing kidney damage.

## Popular scientific summary

- Cod residuals consisting of head, back and skin delayed the increase in blood pressure that is normally seen in obese Zucker fa/fa rats
- Cod residuals did not prevent the development of kidney damage that spontaneously develop in obese Zucker fa/fa rats

High blood pressure is the leading risk factor for cardiovascular and chronic renal diseases (1), and it was estimated that 1.13 billion people had raised blood pressure in 2015 (2). Efficient medical treatment for hypertension is available for those who can afford it; however, the prevention of the development of hypertension should be equally important. Lifestyle modifications are recommended to prevent hypertension in order to avoid premature cardiovascular disease (3, 4). Several intervention and observation studies show a negative association between fish fillet consumption and blood pressure in various population (5,–10), but little is known about whether proteins from fish residuals may affect blood pressure in humans.

It was recently shown that diets containing fillet from Atlantic salmon (a fatty fish) (11), press cake protein powder with high gut content from Atlantic cod (12), or hydrolysed press cake protein powder from headed and gutted blue whiting (a cod-fish) (13) delayed the development of high blood pressure in obese Zucker fa/fa rats. In contrast, diets containing muscle from Atlantic cod (a lean fish) did not affect the blood pressure development in obese Zucker fa/fa rats (12, 14), and the consumption of hydrolysates of muscles from cod, haddock or salmon did not affect blood pressure development in spontaneously hypertensive rats (15). These findings indicate that potent components affecting blood pressure development may be found in lean fish residuals such as skin, viscera, heads and frames.

A blood pressure lowering effect of fish may be mediated through the renin-angiotensin system. Angiotensinogen is cleaved by renin to the biologically inactive angiotensin I, which is then converted to the active vasoconstrictor angiotensin II by angiotensin-converting enzyme (ACE) (16). Motifs with ACE inhibiting activity in vitro have been identified in fish fillet and residuals including skin and backbone (17–20). Other nutrients found in fish may also affect blood pressure. Examples are arginine, which is a precursor for the formation of the vasodilator nitric oxide (21), sodium, which acts as a vasoconstrictor through its control of blood volume by increasing arterial constriction and peripheral vascular resistance (22) and affects renin release (23), and taurine through hitherto unknown mechanisms of action (24–26).

ACE inhibitors were the most commonly used medication for hypertension treatment in the US in 2014 (27) and are also prescribed to delay the progression of chronic kidney disease and amend proteinuria in patients (28–30). In vitro studies have demonstrated an ACE-inhibiting activity of cod residuals containing gut (12), hydrolysed press cake protein powder from headed and gutted blue whiting (13) and hydrolysed fillets from haddock, cod and whiting (15). It is, therefore, of interest that beneficial effects on markers of kidney function were observed in Zucker fa/fa rats fed salmon fillet (11), proteins from cod fillet (31) and proteins from residual materials of herring and salmon (20) but were not affected by proteins from headed and gutted blue whiting (13) or cod fillet (12).

The obese Zucker fa/fa rat presents a range of abnormalities similar to those seen in humans with obesity, including insulin resistance, dyslipidemia, mild glucose intolerance and hypertension (32). The Zucker fa/fa rat is a valuable experimental model for hypertension as it develops an age-related increase in blood pressure, which is also the case for humans (33). These rats are visibly obese from the age of 3–4 weeks and experience an increase in blood pressure already before the age of 10 weeks (32, 34, 35). With increasing age, they also spontaneously develop proteinuria and focal segmental glomerulosclerosis leading to renal failure (36).

Industrial fish processing typically gives 40–50% yield of the main edible product (i.e., fillet), and only minor parts of the protein-rich residuals such as viscera, head, backbone, skin and trimmings are used for human consumption (37). To a large extent, these protein rich coproducts are discarded or used for feed grade fishmeal or fish silage production (38). Documentation of health effects may motivate both consumers and producers to make use of such coproducts in food applications and contribute to improved utilisation of limited fish resources.

The primary objective of the present study was to investigate the effects of 4 weeks intervention with a diet containing proteins from residuals from Atlantic cod, i.e. a mixture of head, backbone and skin (HBS) on the development of high blood pressure in obese Zucker fa/fa rats, compared with Zucker fa/fa rats fed a control diet devoid of fish. The secondary objectives were to investigate any effects of the HBS diet on kidney morphology and markers of kidney function and organ damage, and to examine the in vitro renin and ACE inhibiting properties of the cod residual protein powder and casein. Our hypothesis was that a diet containing HBS from cod would attenuate the development of high blood pressure in obese Zucker fa/fa rats.

## Methods

### Ethical statement

The study protocol was approved by the National Animal Research Authority (Norway) in accordance with the Animal Welfare Act and the Regulation of animal experiments (Approval No. 11603). All applicable international, national and institutional guidelines for the care and use of animals were followed.

### Animals and diets

Twelve male obese Zucker fa/fa rats (HsdHlr:ZUCKER-Leprfa) were obtained from Harlan Laboratories (Indianapolis, IN, USA). The rats were housed in pairs in individually ventilated cages (IVC type 4, blue line from Tecniplast, Buguggiate, VA, Italy) with plastic housing, under standard conditions with a temperature of 23–25°C and a light-dark cycle of 12 h. Rats were acclimatised for a minimum of 7 days under these conditions, before being randomly allocated to the intervention group (HBS) or the Control group by drawing lots, with six rats in each group. The intervention period started when the rats were 8–9 weeks old and weighed 352 ± 8 g (mean ± SD). The number of rats per group was chosen based on previous studies on the development of high blood pressure in obese Zucker fa/fa rats in experimental groups of six rats each (11–13). Rats were fed modified semi-purified diets based on the American Institute of Nutrition’s recommendation for growing laboratory rodents (AIN-93G) (39) with the addition of 1.6 g methionine/kg diet as recommended by Reeves (40) for 4 weeks and differed only in their protein sources (Table 1). Both diets contained 20 wt% of proteins. The AIN-93G diet was used instead of the AIN-93M diet for maintenance containing 15 wt% protein, since rats would be in the growth phase throughout the intervention period (based on growth charts for Zucker fa/fa rats from Harlan Laboratories, https://www.envigo.com). In addition, obese Zucker fa/fa rats have an impaired protein metabolism, which leads to inferior protein utilisation and they therefore requires a higher protein intake to maintain a maximal rate of protein gain during growth (41). Casein was the sole protein source in the Control diet. Cod residual protein powder was added to the HBS diet in an amount providing 25 wt% of total protein, whilst casein constituted the remaining 75 wt% of protein. All ingredients were purchased from Dyets Inc. (Bethlehem, PA, USA) except casein, which was purchased from Sigma-Aldrich (Munich, Germany), and the residual protein powder which was prepared from cod HBS by Nofima (Bergen, Norway).

**Table 1:** Composition of the experimental diets

Contents (g/100g diet)	Control diet	HBS diet
Casein^a^	23.02	17.27
HBS^b^	–	7.64
Cornstarch	49.77	47.88
Sucrose	9.00	9.00
Cellulose	5.00	5.00
Soybean oil	7.00	7.00
t-Butylhydroquinone	0.0014	0.0014
Mineral Mix (AIN-93-MX)	3.50	3.50
Vitamin Mix (AIN-93-VX)	1.00	1.00
L-Methionine	0.16	0.16
L-Cystine	0.30	0.30
Choline bitartrate^c^	0.25	0.25
Growth and maintenance supplement^d^	1.00	1.00

HBS: heads, backbones and skin from cod.

a Contains 86.9% crude protein, 9.0% moisture;

b contains 65.5% crude protein, 4.2% fat, 7.3% moisture, 26.1% ash;

c contains 41% choline;

d contains vitamin B12 (40 mg/kg) and vitamin K1 (25 mg/kg) mixed with sucrose (995 g/kg) and dextrose (5 g/kg).

### Preparation of cod residual protein powder

Atlantic cod (*Gadus morhua*; size 2.6–6.2 kg, *n =* 13) was captured in the Norwegian Sea outside Lofoten, Norway, March 2019 by the industry trawler Granit (Halstensen Granit AS, Bekkjarvik, Norway) and frozen whole on-board at –30°C. The fishes were partly thawed overnight at approximately 18°C, and were manually gutted, headed and filleted. The head was removed by use of an oblique cut behind the pelvic fins in accordance with the procedure applied in the fish processing line on-board the trawler. The skin was removed from the fillet and combined with head and backbone to constitute the HBS fraction. Kidney and heart followed the backbone and head, respectively, and ended up in the HBS-fraction. The HBS-fraction was coarsely ground, mixed and vacuum packed and stored at –20°C before further processing.

The frozen HBS fraction was thawed overnight at 4°C, added water (4:1 on a weight basis), heated to 85°C under continuous stirring and kept at this temperature for 10 min. The heat coagulated raw material was mechanically dewatered in a high-pressure tincture-press. The press liquid was centrifuged to remove excess oil and suspended solids, and the latter (sediment after centrifugation) was combined with the press cake before drying in a fluid bed dryer at 45–50°C. The liquid and fat phases were discarded. The wet press cake was added antioxidants (200 ppm propyl gallate and 20 ppm citric acid on dry matter basis) before the drying operation. The dried press cake powder was milled on a Retsch rotomill (aperture 0.75 mm) and was stored at –80°C until analysis and formulation of the rat diet.

### Design

Rats were fed ad libitum for 4 weeks, with free access to tap water and chewing sticks. Rats were weighed weekly during the intervention period. Blood pressure was measured using the tail-cuff method in conscious rats at baseline (Day 0, before rats were introduced to the experimental diets), on Day 14, and 3 days before endpoint (Day 26). One week before endpoint, rats were housed individually in metabolic cages (Ancare Corp., NY, USA) for 24 h for collection of urine and measurements of water and feed intakes, without fasting in advance. At the end of the experimental period, after a 12 h fast, rats were euthanised whilst anaesthetised with isoflurane (Isoba vet, Intervet, Schering-Plough Animal Health, Boxmeer, The Netherlands) mixed with nitrous oxide and oxygen. Blood was drawn from the heart and collected in BD Vacutainer SST II Advance gel tubes (Becton, Dickinson and Company) for isolation of serum, and serum was frozen at −80°C. The abdominal aorta was isolated through a frontal approach, and the left kidney was perfused using ice-cold PBS by cannulating the distal abdominal aorta and ligating the aorta proximal to the renal arteries. The left kidney was cut in 1–2 mm thick transversal slices and fixed in 4% buffered formaldehyde for morphology. Epididymal white adipose tissue (WATepi) was dissected out and weighed.

The personnel handling the rats and conducting the analyses were blinded to the rats’ group allocation, and rats were handled and euthanised in random order.

### Analyses of diets

Contents of amino acids, fatty acids and energy in diets, and contents of amino acids, total fat, moisture and ash in the fish protein powders were measured by Nofima BioLab (Bergen, Norway). Amino acids were measured by HPLC after hydrolysis in 6N HCl for 22 h at 110°C, using the Waters-Accq-Tag method and fluorescence detection with excitation/emission at 250/395 nm (42). Tryptophan was chemically determined by the method of Miller (43). Dietary caloric content was determined by a bomb calorimeter method in accordance with ISO9831:1998 (44) using a Parr 6400 calorimeter (Parr Instrument Company, Illinois). Fat content was determined gravimetrically after chloroform/methanol extraction (45). Moisture content was measured gravimetrically after drying in a forced-air oven at 103 ± 1°C for 4.5 h (46). The fatty acid compositions of the diets were analysed by gas chromatography (47) after lipid extraction as described by Bligh and Dyer (45). Total ash content was determined gravimetrically after incineration at 550°C (48). Sodium was quantified using inductively coupled plasma optical emission spectrometry. Vitamins A (retinol), D_3_ and E (sum of α-, β-, δ- and γ-tocopherol) in diets were quantified by Eurofins (Moss, Norway) using liquid chromatography with PhotoDiode Array Detection (vitamins A and D_3_) or fluorescence detector (vitamin E).

### Blood pressure measurements

Rats were pre-warmed in a heating cabinet at 32°C for 30 min before blood pressure was measured 10 times using the tail-cuff method (CODA-6, Kent Scientific Corporation, Torrington, CT, USA). Systolic and diastolic blood pressures were measured, and mean arterial pressure (MAP) was calculated as (diastolic blood pressure + 1/3 [systolic blood pressure – diastolic blood pressure]). The same operator performed all blood pressure measurements and was blinded to the rats’ group affiliations. The rats’ blood pressures were measured in randomised order. The blood pressure measurements were conducted in conscious rats, and rats were hand-tame and trained to be in the constrainer before the baseline measurements. The rats were placed in holders on a warming platform (both from Kent Scientific Corporation). Ten cycles with 5 s delay between cycles (without acclimatisation cycles in advance) were measured under close monitoring by the operator, and readings disturbed by the rats’ change of position, sudden movements or flick of the tail were discarded. Max occlusion pressure was 250 mmHg, the deflation time was 15 s and the minimum volume was 15 µL.

### Analyses in serum and urine

Serum concentrations of creatinine, carbamide, alanine transaminase and aspartate transaminase (the latter two were measured with pyridoxal phosphate activation), and urine concentrations of creatinine, total protein, carbamide acid and glucose were analysed on the Cobas c111 system (Roche Diagnostics GmbH, Mannheim, Germany) using the CREP2 (Creatinine plus ver.2), UREAL (Urea/BUN), ALTL (Alanine aminotransferase acc. IFCC), ASTL (Aspartate aminotransferase), TP2 (Total Protein Gen.2 monochromatic) and GLUC2 (Glucose HK) kits from Roche Diagnostics. Sodium in urine was quantified using the Cobas c111 system (Roche Diagnostics GmbH, Mannheim, Germany), equipped with the Ion-Selective Electrode module from Roche Diagnostics. Urine concentration of T cell immunoglobulin mucin-1 (TIM-1) was quantified using the Rat TIM-1/KIM-1/HAVCR Quantikine ELISA kit (cat. no. RKM100) from R&D Systems, Bio-Techne, Minneapolis, MN, USA. A theoretical glomerular filtration rate was estimated as described for rats based on 24 h urine volume and serum and urine concentrations of creatinine and carbamide (49).

As a measure of nitric oxide, the stable metabolites of nitric oxide metabolism, i.e. nitrite and nitrate, were quantified in serum using the Nitrite/Nitrate Assay Kit (cat #23479, Sigma-Aldrich, Munich, Germany) based on the Griess assay. Serum was filtered (Amicon Ultra-0.5 Centrifugal Filter Unit with Ultracel membrane 10K device, Merck KGaA, Darmstadt, Germany) to remove haemoglobin and proteins before analysis of nitrite and nitrate.

### Light microscopy and morphometry of kidneys

Fixed kidney slices were processed by standard procedures and embedded in paraffin. Three-micrometre thick sections were stained with periodic acid Schiff. Slides were scanned with ScanScope® XT (Aperio) at ×40 resulting in a resolution of 0.25 µm per pixel. Virtual slides were viewed in ImageScope v12.4. All microscopic investigations were performed in a blinded manner. Six regions of interest with an edge length of 1,500 µm covering both outer and inner cortex were randomly chosen. All glomeruli in these regions were analysed regarding segmental or global sclerosis (present/absent), and the percentage of podocytes with adsorption droplets (present/absent) was calculated to assess distortion of the filtration barrier. On average, 73 ± 10 glomeruli per rats were examined.

### Renin and ACE inhibition in vitro

Casein and the fish residual protein powder were added Trizma buffer (50 mM, pH 8.0) and hydrolysed using trypsin from bovine pancreas (T1426 from Sigma) at 45°C for 4 h as recommended by Shalaby et al. (50). Proteins in the hydrolysates were quantified on the Cobas c111 system (Roche Diagnostics GmbH, Mannheim, Germany) using the TP2 kit from Roche on the Cobas c111 system. Renin inhibition was measured using the Renin Assay Kit (MAK157, from Sigma-Aldrich) as described in the user manual. ACE-inhibition was measured using the method by Shalaby et al. (50).

### Outcomes

The primary outcome was to investigate the effects of 4 weeks intervention with a diet containing proteins from a mixture of HBS from Atlantic cod on the development of high blood pressure in obese Zucker fa/fa rats. The secondary outcomes were to investigate kidney morphology and markers of kidney function and organ damage and to examine the in vitro renin and ACE inhibiting activities of the cod protein powder.

### Sample size

The present study is, to the best of our knowledge, the first study to investigate the effects of a diet containing proteins from HBS from Atlantic cod on the development of high blood pressure in obese Zucker fa/fa rats. Therefore, data on effect size were not available for sample size calculation or minimally detectable effect sizes for the present study. The study was designed with six rats per group based on previous studies using diets containing proteins from fish on blood pressure development in obese Zucker fa/fa rats showing significant effects with group sizes of six rats (11–13).

### Statistical analyses

Statistical analyses were conducted using SPSS Statistics version 28 (SPSS, Inc., IBM Company, Armonk, NY, USA). All variables were evaluated for normality using the Shapiro–Wilks test, Q–Q plots and histograms. Variables that were not normally distributed underwent log-transformation before parametric statistical tests were performed. The Independent samples *T*-test was used to compare groups. Changes in MAP from baseline to Day 14 and from baseline to Day 26 (endpoint) within each group were tested using the Paired-samples T test, and the within-group changes were compared between the groups using the Independent samples *T*-test and two-way repeated measures ANOVA for three measurements of MAP. The cut off value for statistical significance was set at a probability of 0.05. Results are presented for *n* = 6 rats in each experimental group.

## Results

### Diets, dietary intake and growth

The diets were similar (difference ≤ 20%) in regard to contents of indispensable amino acids and arginine, whereas the sulfonic acid taurine was found only in the HBS diet (Table 2). Analysis of the fatty acid composition of the diets showed few differences between the diets, but small amounts of the long-chain n-3 polyunsaturated fatty acids 20:5n-3 (EPA) and 22:6n-3 (DHA) were found in the HBS diet but not in the Control diet. The complete fatty acid profiles for the experimental diets are presented in Supplemental Table 1. The amounts of vitamins A, D_3_ and E were similar between the diets.

**Table 2:** Dietary content of indispensable amino acids, arginine, taurine, n-3 long-chain polyunsaturated fatty acids and fat-soluble vitamins in the experimental diets, and the in vitro IC50 ACE inhibition by casein and HBS

	Control diet	HBS diet
Amino acids (g/100 g diet)		
Arginine	0.67	0.84
Histidine	0.55	0.54
Isoleucine	1.0	0.95
Leucine	1.9	1.8
Lysine	1.7	1.7
Methionine	0.74	0.69
Phenylalanine	1.1	1.0
Threonine	0.78	0.80
Tryptophan	0.25	0.24
Valine	1.3	1.2
Taurine	ND	0.02
Fatty acids (g/100 g diet)		
20:5 n-3	ND	0.02
22:5 n-3	ND	ND
22:6 n-3	ND	0.04
Fat soluble vitamins (mg/100 g)		
Vitamin A (retinol)	0.14	0.11
Vitamin D3	0.0033	0.0031
Vitamin E^a^	13.5	14.0
IC50 ACE inhibition by dietary proteins (µg/ml)^b^	303 ± 2	422 ± 23*

HBS: heads, backbones and skin from cod; ND: not detected.

Means of two measurements; deviations were <5% between parallels.

a Vitamin E: sum of α-, β-, δ- and γ-tocopherol;

b Data are presented as the amount of protein in µg/mL needed to inhibit 50% of the ACE activity in a 0.25 U ACE assay, and are presented as the mean with their standard error of mean shown for two measurements. Groups are compared using the Independent samples *T*-test; *P* < 0.05 was considered significant. The asterisk indicates a significant difference between the dietary proteins tested.

For renin in vitro activity inhibition, no measurable inhibition was detected for any of the dietary proteins. The capacity for in vitro inhibition of ACE activity was significantly less potent for HBS compared to casein (i.e. higher IC50 concentration, *P* 0.0072, Table 2).

The bodyweight at baseline, the percent growth from baseline to endpoint, the weight of WATepi relative to bodyweight and the daily energy and protein intakes measured in week 3 were similar between groups (Table 3). The water intake (g/kg bodyweight/24 h) and urine volume (g/kg bodyweight/24 h) were similar between the groups, whereas the sodium intake from feed was higher in the HBS group compared to the Control group (Table 3).

**Table 3:** Bodyweight, growth, relative weight of WATepi, dietary intake, water intake and urine volume (means and standard deviations)

	Control group	HBS group	P
Bodyweight (g) at baseline	353 ± 10	353 ± 5	0.94
Growth (% change in bodyweight from baseline to endpoint)	48 ± 12	56 ± 5	0.11
WATepi (g/kg BW)	31 ± 3	32 ± 2	0.72
Energy intake (kJ/kg BW/24 h)	954 ± 102	1,024 ± 107	0.27
Protein intake (g/kg BW/24 h)	11.0 ± 1.2	12.2 ± 1.3	0.13
Sodium intake (mg/kg BW/24 h)	57 ± 6	79 ± 8	0.00034
Water intake (g/kg BW/24 h)	49.2 ± 17.7	42.7 ± 12.7	0.83
Urine volume (g/kg BW/24 h)	38.8 ± 16.9	34.5 ± 8.2	0.59

Data are presented as mean ± standard deviation for *n* = 6 rats in the Control group and *n* = 6 rats in the HBS group. *P* values are shown for the comparisons of the Control group and the HBS group using the Independent samples *T*-test; *P* < 0.05 was considered significant; HBS: heads, backbones and skin from cod; WATepi: epididymal white adipose tissue.

### Blood pressure

**Fig. 1:**
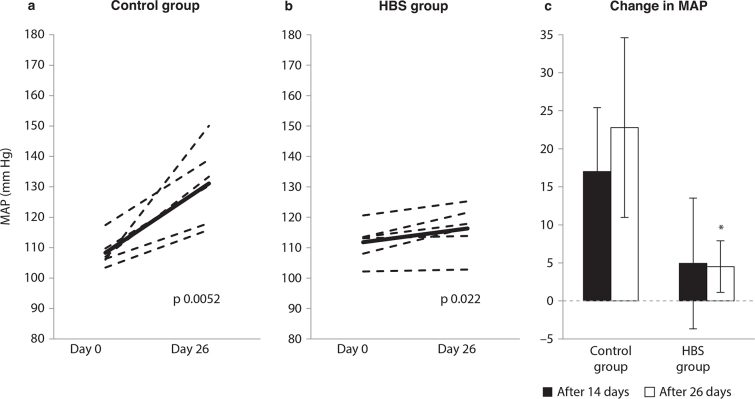
Mean arterial blood pressure (MAP) at Day 0 and Day 26 in the Control group (A) and the HBS group (B), presented for individual rats (broken lines) with means (bold lines) and compared using the Paired-samples T test. The changes in MAP from Day 0 to Day 14 (black bars) and from Day 0 to Day 26 (white bars) are presented as mean with their standard deviation shown by vertical bars (C). Groups are compared using the Independent samples *T*-test; *P* < 0.05 was considered significant. The asterisk in (C) indicates significant differences between the groups after 26 days. The results are presented for *n* = 6 rats in the Control group and *n* = 6 rats in the HBS group. HBS: heads, backbones and skin fraction.

The MAP was similar between the groups at baseline (*P* 0.31). MAP was significantly increased from Day 0 to Day 14 in the Control group (*P* 0.0053) but was unchanged in the HBS group (*P* 0.43), and *P* for comparison of within-group changes was 0.10 (Fig. 1C). After 4 weeks, MAP was significantly increased in both the Control group and the HBS group relative to baseline MAP (Figs. 1A–B), and the increase in MAP from baseline to endpoint was significantly less pronounced in the HBS group when compared to the Control group (*P* 0.0045, Fig. 1C). A repeated measures ANOVA analysis for measurements of MAP at the three time-points revealed significant differences between the two groups (*P* 0.020).

### Morphological examinations of kidneys

The percentage of glomeruli with absorption droplets was similar between the HBS group and the Control group (Table 4). Segmental sclerosis was present only in one glomerulus in the Control group. Global glomerulosclerosis and tubular atrophy were not detected in any of the groups.

**Table 4:** The percentage of glomeruli with absorption droplets, markers of kidney function and organ damage, and serum concentration of nitrite + nitrate (means and standard deviations)

	Control group	HBS group	P
Percentage of glomeruli with absorption droplets	29.2 ± 22.1	15.3 ± 13.5	0.22
Serum creatinine (µmol/L)	12.1 ± 1.9	13.0 ± 2.6	0.49
Serum carbamide (mmol/L)	5.0 ± 0.9	6.0 ± 1.0	0.075
Serum alanine transaminase (U/L)	114 ± 39	134 ± 45	0.42
Serum aspartate transaminase (U/L)	248 ± 88	248 ± 99	1.00
Serum nitrite + nitrate (ng/µL)	0.29 ± 0.16	0.43 ± 0.19	0.22
Urine creatinine (mmol/L)	3.8 ± 1.4	4.3 ± 1.1	0.46
Urine total protein (g/mmol creatinine)	2.1 ± 1.0	1.8 ± 1.0	0.61
Urine carbamide (mmol/mmol creatinine)	377 ± 26	350 ± 42	0.20
Urine TIM-1 (ng/mmol creatinine)	308 ± 98	222 ± 43	0.078
Urine glucose (mmol/mmol creatinine)	5.7 ± 9.3	0.7 ± 4.5	0.082
Urine sodium (mmol/mmol creatinine)	18 ± 2	22 ± 3	0.011
Estimated GFR (mL/24 h)	10,482 ± 2,068	9,918 ± 2,525	0.68

Data are presented as mean ± standard deviation for *n* = 6 rats in the Control group and *n* = 6 rats in the HBS group. *P* values are shown for the comparisons of the Control group and the HBS group using the Independent samples *T*-test; *P* < 0.05 was considered significant; HBS: heads, backbones and skin from cod; TIM-1: T cell immunoglobulin mucin-1; GFR: glomerular filtration rate.

### Markers of kidney function and organ damage

At endpoint, the serum concentrations of creatinine, carbamide, alanine transaminase, aspartate transaminase and nitrite + nitrate were not significantly different between the experimental groups (Table 4). The groups were also similar with regard to the urine creatinine concentration and the concentrations (relative to creatinine) of total protein, carbamide and the estimated glomerular filtration rate. The urine sodium concentration (relative to creatinine) was significantly higher in the HBS group, and there were tendencies (*P* < 0.1) for lower urine concentrations (relative to creatinine) of glucose and TIM-1 in HBS fed rats when compared to the Control group.

## Discussion

In the present study, we show for the first time that a residual protein powder consisting of head, backbone and skin from Atlantic cod attenuated the development of high blood pressure in obese Zucker fa/fa rats. Morphological examinations of the kidney and biochemical analyses of urine revealed that the effect on blood pressure in the HBS group was not accompanied with less pronounced kidney damage when compared to the Control group.

Young obese Zucker fa/fa rats, aged around 9 weeks at the start of the intervention, were used in this study, since these rats spontaneously develop an increase in blood pressure before the age of 10 weeks (32, 34, 35) and develop proteinuria and focal segmental glomerulosclerosis, ultimately leading to renal failure as they get older (36). The rats in the Control group experienced an increase in MAP that was evident already after 2 weeks intervention and MAP continued to increase to the 4-week measurement. Contrary to this, no change was seen in MAP after 2 weeks in the HBS group. After 4 weeks, the increase in MAP was significantly smaller in the HBS group compared to the Control group, thus demonstrating a potential for cod residuals to attenuate the development of high blood pressure in this rat model. This is in line with recent findings in studies using codfish; diets containing protein powder from cod residuals (12) or headed and gutted blue whiting (13) delayed blood pressure increase in obese Zucker fa/fa rats. These findings together support our assumption that residual materials from cod may contain components of significant value for the prevention of high blood pressure.

Blood pressure is controlled by several mechanisms, of which the renin-angiotensin system may be the best known. Renin and ACE are important enzymes for blood pressure control as they are involved in catalysing the conversion of angiotensinogen via angiotensin I to the vasoconstrictor angiotensin II (16). Consequently, peptides with renin or ACE inhibiting properties may lower blood pressure by reducing the production of angiotensin II. Motifs with ACE inhibiting activities have been identified in muscles and residuals from fish (17–20), and in vitro inhibition of ACE and/or renin activities has been observed for proteins from muscles, viscera, skin and other residuals from several fish species including cod (12, 13, 15, 19, 20, 51, 52). However in the present study, after trypsin digestion, neither HBS nor casein inhibited the in vitro renin activity, and the ACE IC50 value of the HBS protein powder was significantly higher (i.e. less potent) when compared to casein and did, therefore, not correspond to the observed effects of the diets on blood pressure. These in vitro assessments of renin and ACE inhibitory activities are not sufficient for concluding whether the attenuated blood pressure increase in rats fed the HBS diet may or may not involve the ACE pathway, since the peptides produced during in vivo digestion may differ significantly from those liberated by trypsin digestion.

Components other than the proteins in the diets may have influenced the blood pressure development in the rats in this study. Taurine was detected in the HBS diet but not in the Control diet. Although the taurine content in the HBS diet was quite low, this is of interest since taurine has been shown to have a blood pressure lowering effect in both humans and rats (24–26). The mechanisms behind this are not fully elucidated, but studies in isolated rat heart myocytes suggest that taurine reverses the action of angiotensin II by altering the Ca^2+^ flux across cell membranes (53). Another interesting aspect of taurine is its ability to act as an antioxidant (54) since oxidative stress can inactivate the vasodilator nitric oxide and, thus, contribute to generate and maintain hypertension (21, 55). The obese Zucker fa/fa rat presents evidence of oxidative stress (56), which may have a role in the development of high blood pressure and renal damage in this strain (34). However, the dietary contents of antioxidant vitamins A, D_3_ and E were similar between the HBS diet and the Control diet and can, therefore, not explain the differences in blood pressure development between the groups. In addition, several studies have indicated that there is an inverse association between the intake of fatty fish or fish oil supplements and blood pressure in both humans (6, 57, 58) and rats (11, 59, 60). Although the content of long chain n-3 polyunsaturated fatty acids was low in the cod residual diet, these fatty acids may have contributed to the beneficial effects of cod residuals on blood pressure development.

Dietary supplementation of arginine has been shown to lower blood pressure in humans (61), probably by serving as a substrate for vascular production of the vasodilator nitric oxide (21). Arginine from fish has been suggested to be an important contributor to the delayed development of hypertension in spontaneously hypertensive rats fed fish proteins (62). The content of arginine showed little variance between the diets in the present study, and the serum nitrite + nitrate concentration (as a measure of nitric oxide) was similar between the groups. Therefore, it is not likely that dietary arginine affected the blood pressure development in the present study. Despite the slower increase in blood pressure in the HBS group, the dietary sodium intake was higher with no differences between the groups for water intake and urine volume. Although rats seemed to efficiently excrete excess sodium in urine, it is possible that the effects on blood pressure would have been more prominent if the dietary sodium content in the HBS diet was lower.

The obese Zucker fa/fa rat develops proteinuria already at around the age of 10 weeks (36). At this stage, due to increased glomerular intravascular pressure, the glomerular epithelial cells (podocytes) might show signs of damage such as absorption droplets or pseudocysts (63). The rats in the present study were 12–13 weeks old at the end of the intervention, and the morphological investigation revealed a similar percentage of absorption droplets in kidneys from the two experimental groups. However, kidneys did not show any signs of atrophic changes or segmental sclerosis in glomeruli in any of the rats. Protein was found in urine from all rats, thus indicating renal damage (64) in both groups. The urine protein concentration (relative to creatinine) and the estimated glomerular filtration rate (actual glomerular filtration rate was not measured) were similar between the HBS group and the Control group. In addition, we found no differences between the groups for serum concentration of creatinine, a commonly used marker of kidney dysfunction (65), or for relative urine concentrations of TIM-1, which is an indicator of tubular injury (66), and glucose, which is indicative of proximal tubule damage leading to reduced reabsorption of glucose. The dietary groups also had similar serum concentrations of organ function markers (alanine transaminase and aspartate transaminase). Thus, based on the current findings, 4 weeks intervention with the HBS diet seems not to be sufficient to protect young obese Zucker fa/fa rats against renal damage at this early stage of development of decreased renal function.

The present study has some methodological strengths and limitations. This study was designed to investigate the effects of a diet containing protein powders made from residuals, i.e. head, backbone and skin from cod, on the development of high blood pressure and renal damage, and the experimental diet is relevant for human nutrition. Although the spontaneously hypertensive rat is the most commonly used rat model for studies on the development and treatment of hypertension, this rat is representative of only a rare subtype of human hypertension; primary hypertension that is inherited in a Mendelian fashion (67). We therefore investigated the effects of cod residuals in obese Zucker fa/fa rat, which develops an age-related increase in blood pressure as is also seen in humans (33), since this may be a more relevant model where hypertension coexists with obesity and other metabolic disturbances in a real-life setting (35). The protein-rich HBS powder was investigated as potential protein supplement for human consumption, and not as a meal replacement. Future studies should investigate if these findings are relevant for human hypertension and renal function. Blood pressure measurements were conducted in conscious rats using the tail-cuff method (volume-pressure recording). The tail-cuff method is a non-invasive and inexpensive method that does not require surgery and was chosen instead of continuous intravascular blood pressure measured by telemetry since the comparison of these methods shows similar results over the physiological range of blood pressure in mice (68). Rats were hand-tame and trained to be in the constrainer before the start of the intervention. To reduce the number of animals, and in line with the 3Rs, we did not include lean Zucker rats since they do not experience an increase in blood pressure as they age, at least not in the age span relevant to the present study. Data on effect size were not available for sample size calculation or minimally detectable effect sizes for the present study. Consequently, we designed the present study with six rats per group based on studies on effects of diets containing proteins from fish on blood pressure development in obese Zucker fa/fa rats, showing significant effects with group sizes of six rats (11–13). The present study is small but will constitute a base for sample size calculations for future studies with similar designs. Assessments of renin and ACE inhibitory activities of the cod protein powders by using an in vitro assay are not sufficient for concluding whether the lower blood pressure increase observed in vivo is mediated through the renin-angiotensin system. A direct measurement of albuminuria and glomerular filtration rate would strengthen the assessment of kidney function, but this was not feasible in the current study.

To conclude, findings in this study demonstrate that the HBS diet effectively delayed the increase in blood pressure that is typically observed in obese Zucker fa/fa rats. The attenuation of the increase in blood pressure in rats fed the HBS diet was not accompanied with less kidney damage or better kidney function. Future studies should identify the fractions or components in fish residuals that affect blood pressure development in animal models and investigate the effects of these components on blood pressure in humans with increased risk of developing hypertension.

### Ethical approval

All applicable international, national and institutional guidelines for the care and use of animals were followed. All procedures performed in studies involving animals were in accordance with the ethical standards of the institution or practice at which the studies were conducted.

## Supplementary Material

Intake of residuals from Atlantic cod attenuated blood pressure increase but did not delay development of kidney damage in obese Zucker fa/fa rats

## Bibliografical note

Iselin Vildmyren received a PhD in nutrition from the University of Bergen. Her research is centered on health effects of fish residual intake in rats and humans.

Åge Otethals, PhD, is a senior researcher at Nofima AS. He is an expert in feed and food technology, process development and optimisation, decontamination, protein hydrolysis and extrusion technology.

Sabine Leh, PhD, MD, is a pathologist and renal physiology specialist at Haukeland University Hospital and an associate professor at the Universiy of Bergen.

Tor Andreas Samuelsen, PhD, is a researcher at Nofima AS and works with feed and food technology and process development and optimisation.

Alfred Halstensen, PhD, MD, is a professor at the University of Bergen.

Hans-Peter Marti, PhD, MD, is a professor at the University of Bergen.

Oddrun A. Gudbrandsen, PhD, is a professor and nutrition researcher at the University of Bergen. She is a group leader of the Dietary Protein Research Group Gudbrandsen’s research is focusing on health effects of dietary proteins on risk factors for the development of type 2 diabetes and coronary heart disease.
