# Supplementary material for: Intake of residuals from Atlantic cod attenuated blood pressure increase but did not delay development of kidney damage in obese Zucker fa/fa rats
Source: Food Nutr Res. 2022 Oct 11;66:10.29219/fnr.v66.8708. doi: 10.29219/fnr.v66.8708 (PMC9602205; doi:10.29219/fnr.v66.8708)
Supplement: Intake of residuals from Atlantic cod attenuated blood pressure increase but did not delay development of kidney damage in obese Zucker fa/fa rats [file FNR-66-8708-s001.pdf]

Supplemental Table: Fatty acids in the experimental diets

	Control Diet	HBS diet
Fatty acids (g/100g diet)		
14:0	0.03	0.02
16:0	0.84	0.93
18:0	0.27	0.30
20:0	0.02	0.02
22:0	0.02	0.03
16:1n-7	0.01	0.02
18:1 (n-9)+(n-7)+(n-5)	1.60	1.73
20:1 (n-9)+(n-7)	0.02	0.04
22:1 (n-11)+(n-9)+(n-7)	0.01	0.01
24:1n-9	ND	ND
16:2n-4	ND	ND
16.3n-4	ND	ND
18:2n-6	3.73	4.07
18:3n-6	ND	ND
20:2n-6	ND	ND
20:3n-6	ND	ND
20:4n-6	ND	ND
22:4n-6	ND	ND
18:3n-3	0.45	0.49
18:4n-3	ND	ND
20:3n-3	ND	ND
20:4n-3	ND	ND
20:5n-3	ND	0.02
21:5n-3	ND	ND
22:5n-3	ND	ND
22:6n-3	ND	0.04
Unidentified fatty acids	0.22	0.23

HBS: heads, backs and skin from cod; ND: not detected.

Means of two measurements; deviations were <5% between parallels.
